# Correlative confocal and scanning electron microscopy of cultured cells without using dedicated equipment

**DOI:** 10.1016/j.xpro.2021.100727

**Published:** 2021-08-09

**Authors:** Javier Casares-Arias, Miguel A. Alonso, Álvaro San Paulo, María Ujué González

**Affiliations:** 1 Centro de Biología Molecular Severo Ochoa, Consejo Superior de Investigaciones Científicas and Universidad Autónoma de Madrid, 28049 Madrid, Spain; 2 Instituto de Micro y Nanotecnología, IMN-CNM, CSIC (CEI UAM+CSIC), Tres Cantos, Madrid 28760, Spain

**Keywords:** Cell Biology, Microscopy

## Abstract

This protocol enables correlative light and electron microscopy (CLEM) imaging of cell surface features without using dedicated equipment. Cells are cultured and fixed on transparent substrates for confocal microscopy imaging. No conductive coating is employed in the scanning electron microscopy workflow, providing a clean cell surface observation, with fiducial markers assisting alignment of optical and topographical images. This protocol describes CLEM imaging for midbody remnants in MDCK cells but can also be applied to different cell types and surface features.

For complete details on the use and execution of this protocol, please refer to Casares-Arias et al. (2020).

## Highlights

• A CLEM protocol without dedicated equipment requirements
• Confocal and SEM data sets are acquired on independent setups
• Large-scale sample features are used for initial correlation and navigation
• Gold nanobeads are used as fiducial markers during final image alignment

## Graphical abstract

**Figure:**
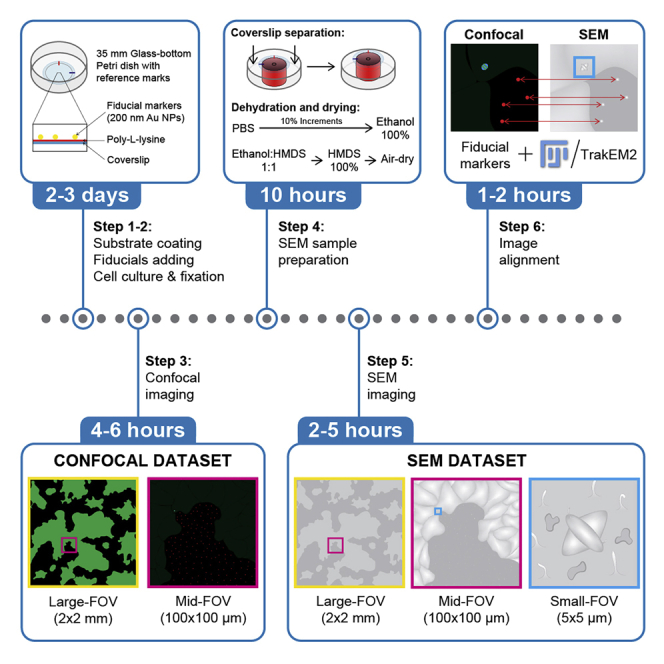

## Before you begin

To be identified under the confocal microscope, the biological structures must be labeled with fluorescent molecules prior to imaging. Immunofluorescence labeling protocols, widely used in the field, usually include a permeabilization step that leads to ultrastructural detail loss, rendering the use of antibodies unsuitable for a CLEM approach. Therefore, in order to localize and analyze the structures of interest, fluorescent fusion proteins or similar approaches, such as click chemistry (SNAP/HALO-tag), must be used.

This protocol can be applied to different cell types and surface features. In the examples included to illustrate the protocol, a MDCK cell line stably expressing two different fluorescent fusion proteins has been used. For further details, please refer to (Casares-Arias et al., 2020).

## Key resources table

**Table:** 

REAGENT or RESOURCE	SOURCE	IDENTIFIER
**Chemicals, peptides, and recombinant proteins**		

Minimum Essential Media (MEM)	Thermo Fisher Scientific	Cat#31095-029
Trypsin	Thermo Fisher Scientific	Cat#27250-018
EDTA (Tritiplex III)	Merck	Cat#108418.0250
Poly-L-Lysine	Merck	Cat#P1524
G418	Santa Cruz	Cat#Sc-29065B
Phosphate Buffer Solution 1 M pH 7.4	Merck	Cat#P3619
Paraformaldehyde	Merck	Cat#30525-89-4
Glutaraldehyde	EMS	Cat#16220
Ethanol	Merck	Cat#1.00983
Acetone	Merck	Cat#24201-M
Hexamethyldisilazane (HMDS)	Merck	Cat#440191
250 nm Gold nanoparticles	BBI Solutions	Cat#em.gc250

**Experimental models: Cell lines**		

MDCK cell line	ATCC	Cat#CRL2936

**Recombinant DNA**		

mCherry-Tubulin	Takara Bio	N/A
pNG72-GFP-L-CHMP4B	Juan Martín Serrano, King’s College London	(Ventimiglia et al., 2018)

**Software and algorithms**		

FIJI-ImageJ2	(Rueden et al., 2017)	imagej.net/ImageJ2
TrakEM2	(Cardona et al., 2012)	https://www.ini.uzh.ch/∼acardona/trakem2.html

**Other**		

35 mm glass-bottom dishes	MatTek	Cat#P35G-1.5-20-C
A1R+ confocal microscope	Nikon	N/A
3M™ Copper Conductive Tape, Single Adhesive Surface	Ted Pella, Inc.	Cat#16072
3M™ XYZ Axis Tape, Electrically Conductive, Double Sided, 9712	Ted Pella, Inc.	Cat#16081
Large Sample Stub for SEM, ∅32 mm	Ted Pella, Inc.	Cat#16148
Field Emission SEM Verios 460	FEI (now Thermo Scientific)	N/A

## Materials and equipment

**Table:** 1× fixing solution

Reagent	Final concentration	Amount
Paraformaldehyde (8% in water)	2%	2.5 mL
Glutaraldehyde (8% in water)	2%	2.5 mL
Phosphate Buffer 1M pH 7.4	0.1 M	1 mL
ddH_2_O	n/a	4 mL
**Total**	**n/a**	**10 mL**

1× Fixing solution can be stored at room temperature (20°C–22ºC) for up to a month.

**Table:** 2× fixing solution

Reagent	Final concentration	Amount
Paraformaldehyde (16% in water)	4%	2.5 mL
Glutaraldehyde (16% in water)	4%	2.5 mL
Phosphate Buffer 1M pH 7.4	0.2 M	2 mL
ddH_2_O	n/a	3 mL
**Total**	**n/a**	**10 mL**

2× Fixing solution can be stored at room temperature (20°C–22ºC) for up to a month.

### Confocal microscopy

Images were captured using a 60× water objective (NA 1.2) on a Nikon A1R+ confocal microscope. 488 and 561 nm laser lines were used to image the fluorescent fusion proteins, 640 nm laser line was used on reflection mode to image the gold nanoparticles (Au NPs). Specific acquisition parameters for each image set are detailed on the step-by-step method details section.

***Note:*** Additional tracks, such a transmitted light, can also be acquired during confocal imaging.

### Scanning electron microscopy

Scanning electron microscopy (SEM) images were acquired with a Field Emission SEM FEI (now Thermo Scientific) Verios 460. High-end recent SEMs, as this model, are able to provide high resolution in the very low voltage (VLV) range (≤ 1 nm at 1 kV). This has allowed us to image uncoated biological samples on glass substrates.

***Note:*** Though we strongly recommend the use of VLV-SEM to avoid the need of coating the samples with conductive layers, the protocol can be adapted to allow the utilization of more conventional SEM equipment (see troubleshooting 1: Using Alternative SEM without VLV capabilities).

The imaging of uncoated biological samples has the advantage of reducing possible artifacts in the observed morphology due to the deposited layer. However, the reduced conductivity of the samples induces charging effects that degrade the image quality and therefore have to be minimized. The use of VLV is a first strategy to achieve this. Additional methods include the use of very short dwell times (50–100 ns). To maximize the signal to noise ratio in these conditions, frame integration scanning modes need to be used (around 100–200 frames per image are recommended), with software-based drift correction option activated (if this option is not available, charge-originated drift may destroy the quality of image after frame integration). Particular acquisition parameters for each image set are specified on the step-by-step method details section.

Most SEM systems providing high resolution at very low voltages have implemented the option of applying beam deceleration (Zarraoa et al., 2019). This method consists in applying a negative bias voltage (typically 0.5–4 kV) to the sample holder in order to decelerate the incident beam before reaching the sample surface. As a consequence, the effective beam landing energy equals the column acceleration voltage (which is kept relatively high −from 2 to 5 kV− for optimum column performance and resolution) minus the sample bias. The resulting electric field between the pole piece and the sample acts as an additional electrostatic lens that reduces the beam diameter and enhances secondary electron (SE) collection at the detector placed inside the column –also called in-lens detector– improving spatial resolution. The SE and back-scattered electrons are also spatially redistributed by this electric field and the amount of SE more pathologically affected by charge effects that are collected by the detector is reduced. Therefore, the use of beam deceleration can improve both the image resolution and the charge management.

## Step-by-step method details

### Coating and addition of gold fiducial markers to the coverslip

**Timing: 2 h**

**Figure 1:**
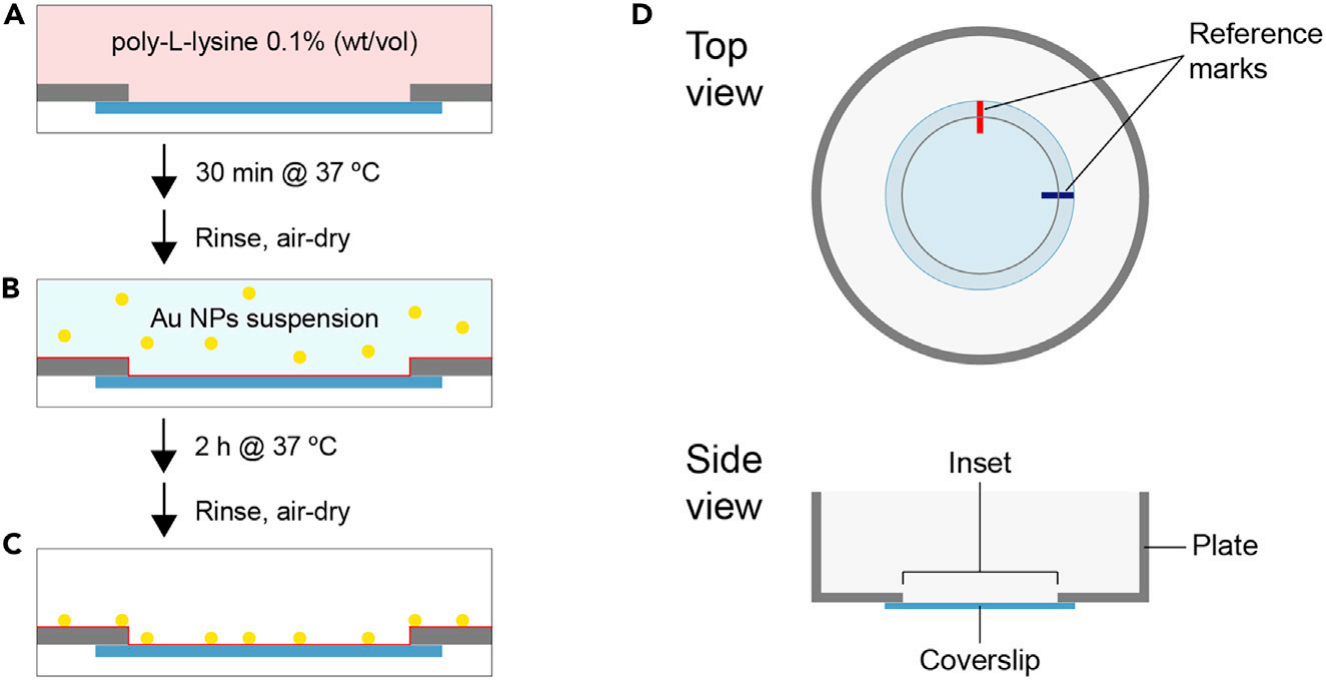
Coverslip coating and fiducial markers attachment procedure First, the coverslip is coated with Poly-L-lysine (A), followed by incubation with a Au NPs suspension (B) and then air-dried (C). Once the surface is ready for cell culture, reference marks are added to the bottom of the coverslip for further alignment (D).

***Note:*** On the presented example, poly-L-lysine has been used only to ensure correct adsorption of the Au NPs to the glass surface, since MDCK cells do not require any substrate coating to grow. Though this possibility has not been tested, alternative coatings required to ensure cell attachment should not interfere with the protocol, provided that they allow the correct visualization of the Au NPS over the substrate.

1. Cover the surface of the inset of a glass-bottomed 35 mm Petri dish with 650 μL of 0.1% (wt/vol) poly-L-lysine solution (dissolved in water) and incubate for 30 min at 37°C (Figure 1A).

2. Rinse with deionized water and air-dry.

***Note:*** Though sterile working conditions are optional during this phase, drying steps should be performed in a fume hood to keep the coverslip surface as clean as possible.

3. Prepare a suspension of Au NPs (in deionized water) to achieve a final density along the coverslip surface of around 1–5 × 10^4^ particles mm^−2^.
  a. Thoroughly vortex the Au NPs stock before pipetting the required volume on a separate tube.
  b. Sonicate the suspension in a bath sonicator for 3 cycles of 30 s at the highest output setting to break up NP aggregates.
  c. Dilute the suspension to the desired concentration in deionized water.

***Note:*** In our case, 250 nm spherical gold nanoparticles were used, as they can be easily observed both under the confocal and scanning electron microscopes. Au NPs with smaller sizes or different shapes can also be used, though final density may need to be adjusted to ensure that individual NPs can be observed near the structures of interest.

**CRITICAL:** The presence of salts in the Au NPs suspension could cause undesired aggregation.

4. Add 650 μL of Au NPs suspension to the inset, incubate for 2 h at 37°C (Figure 1B).

***Note:*** You may need to optimize the final density of fiducial markers by adjusting Au NPs concentration and/or incubation time. Au NPs suspension volume may vary for different dish sizes.

5. Retire the remaining solution and air-dry (Figure 1C).
6. Mark the bottom of the coverslip with an ethanol-resistant marker to orient the sample during subsequent image acquisition steps. Two marks, defining “north” and “east” of the sample are typically enough (Figure 1D).

### Cell culture

**Timing: 2–3 days**

**CRITICAL:** These steps should be carried out under sterile working conditions.

***Note:*** This protocol has been optimized for the imaging of midbody remnants on renal epithelial MDCK cells, other cell types or structures of interest may require additional steps or different conditions.

7. Sterilize the dish previously coated with Au NPs by exposing it to the germicide lamp of a cell culture hood for 15–30 min.
8. Add 2 mL of MDCK cells expressing the desired fluorescent fusion proteins (7.5 × 10^4^ mL^−1^) suspended in MEM to the plate.

**CRITICAL:** The initial number of cells must be optimized according to the cell line and the duration of the culturing phase, so that the culture is not completely confluent by the time of fixing.

***Note:*** Culture media may vary for different cell types.

9. Culture the cells for 48–72 h at 37°C in an atmosphere of 5% CO_2_.

**Figure 2:**
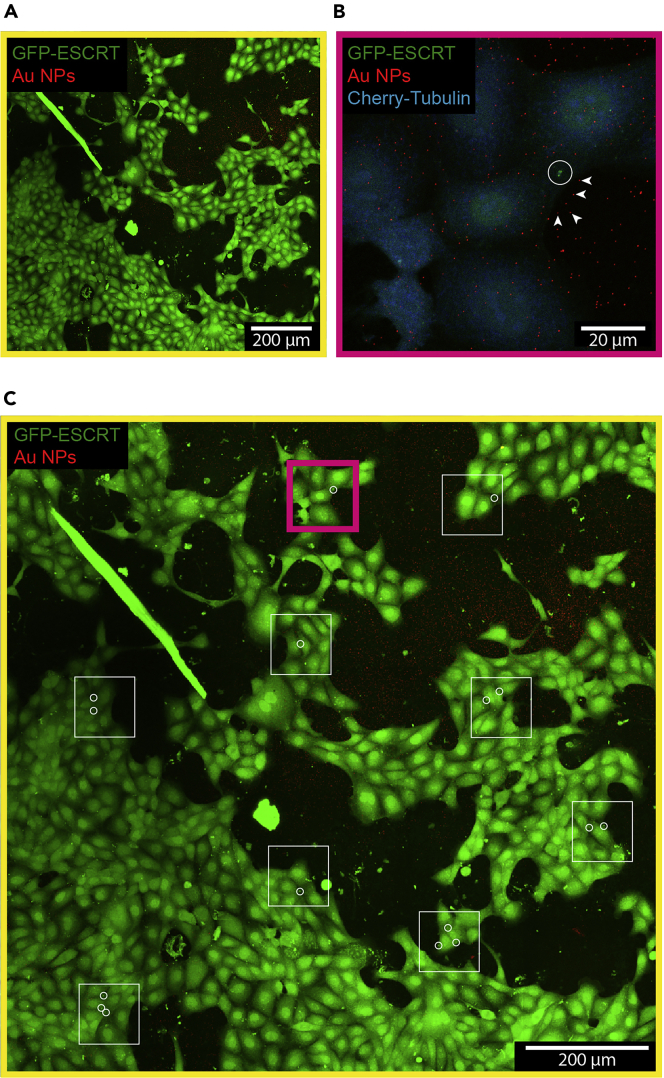
Confocal dataset of the CLEM procedure (A) Confocal large-FOV image (contrast enhanced). (B) Confocal medium-FOV image. (C) Look-up image showing the localization of the medium-FOV images and structures of interest (squares and circles respectively). Arrowheads in (B) show the position of the Au NPs that are closer to the structure of interest (circle). Magenta box in C corresponds to the imaging area shown in B.

**CRITICAL:** For the alignment strategy to work, culture must be subconfluent at the time of fixing. For this, the initial number of cells and duration of the culturing phase must be adjusted. The goal is not to achieve a specific percentage of confluence, but to ensure that the substrate and Au NPs are exposed in some areas (see Figure 2). If the use of subconfluent cultures represents a limitation, see troubleshooting 2: Use of confluent cultures.

### Fixation

**Timing: 2–10 h**

**CRITICAL:** Only electron microscopy-rated fixatives must be used, common-use aldehydes might include traces of organic solvents, such as methanol, that can damage the cell surface.

10. Add a volume of 2× fixing solution (4% paraformaldehyde + 4% glutaraldehyde in phosphate buffer 0.2 M) equal to the volume of culture medium (MEM) in the dish. Incubate for 10 min at room temperature (RT; 20°C–22°C).
11. Remove most of the liquid and add fresh 1× fixing solution (2% paraformaldehyde + 2% glutaraldehyde in phosphate buffer 0.1 M). Incubate for 2 h at 20°C–22°C or 8–10 h at 4°C.

**Pause point:** Samples can be stored in fixing solution at 4°C for up to a week.

**Alternative:** Different storage solutions, such as PBS, can also be used. In such case, the addition of antibacterial agents (Penicilin-Streptomycin or sodium azide) is advised.

### Confocal imaging

**Timing: 4–6 h**

During this phase, the localization of the surface features of interest labeled with fluorescent proteins and the Au NPs present in the sample is determined by confocal fluorescence and reflection microscopy, respectively. Candidate structures for CLEM analysis are selected, and all the relevant confocal images are acquired.

12. Substitute fixing solution with phosphate buffer 0.1 M.
13. Place the dish on the sample stage, using the marks added during step 6 as a reference. Perform the imaging at the center of the coverslip if possible.
14. Acquire a large field-of-view (FOV) (2 × 2 mm) image for alignment and navigation purposes, including the fluorescence signals and the reflection channel showing the Au NPs. In our case, these images were generated by acquiring a 10 × 10 tilescan z-stack (5 μm total with 250 nm steps) with a resonant scanner, 8× averaging and pixel size of 200 nm (Figure 2A).

***Note:*** Acquisition settings must be optimized depending on the abundance of the structure of interest, the proportion of cells expressing the fluorescent protein and signal strength.

**CRITICAL:** We strongly recommend performing all the confocal imaging in one session to guarantee a fixed sample orientation. If this is not possible, the rest of the images can be acquired on a different session, though alignment might be an issue and require special attention.

15. Localize candidate surface features and acquire medium-FOV (100 × 100 μm) images, including the fluorescence signals and the reflection channel showing the Au NPs. We acquired these images with a unidirectional galvano scanner, no averaging and 50 nm pixel size (Figure 2B).

***Note:*** The closer a candidate structure is to an area with exposed substrate, the easier will be to localize it under the scanning electron microscope (SEM).

**CRITICAL:** When selecting the imaging region, be sure to include an area with exposed substrate including Au NPs near the structure(s) of interest (see arrowheads on Figure 2B).

16. As medium-FOV images are recorded, keep track on their position relative to the large-FOV image. This information is needed for the elaboration of the look-up map (see next step). A convenient way of doing this is by using the option that most microscope manufacturers offer of re-centering the stage to a given position of a previously acquired image when a motorized stage is available. During this process, the position of each medium-FOV image is generally annotated on an additional copy of the large-FOV image. If this capability is not available, see “troubleshooting 3: Confocal dataset tracing” for alternatives.
17. Prior to sample removal, record the objective position, and thus imaging area, relative to the coverslip. For this, a picture of the stage adaptor during the imaging session can be taken. Additionally, once the backside of the coverslip has been cleaned, make an additional ethanol-resistant mark on the same position to frame the imaged area.

***Note:*** The mark indicating the area imaged by confocal microscopy is used as starting point for SEM imaging, and thus should be as accurate as possible.

**Pause point:** Samples can be stored for up to a week at 4°C upon substitution of the phosphate buffer by 1× fixing solution.

**Alternative:** Different storage solutions, such as PBS, can also be used. In such case, the addition of antibacterial agents (Penicilin-Streptomycin or sodium azide) is advised.

### Look-up map elaboration

**Timing: 30 min**

In order to facilitate the correlation process, it is recommended to elaborate a look-up map prior to the SEM imaging session. This consists of the large-FOV confocal image of the full area where the position of each medium-FOV imaged is indicated. This will be helpful both for navigation and identification of individual structures in the sample along the SEM session.

18. Open the large-FOV image in ImageJ. Given the file size, performing a z-projection and creating a compressed jpeg version is advised.
19. Highlight and identify the regions that correspond to each medium-FOV image, repeat the process with the candidate structures present inside them. This will allow to identify each structure, keeping track of its corresponding medium and large-FOV images (Figure 2C).

### Coverslip extraction

**Timing: 5 min**

**CRITICAL:** This process involves removing most of the liquid from the dish, it must be carried out as fast as possible to avoid uncontrolled drying.

***Note:*** This phase must be carried out on a fume hood.

**Figure 3:**
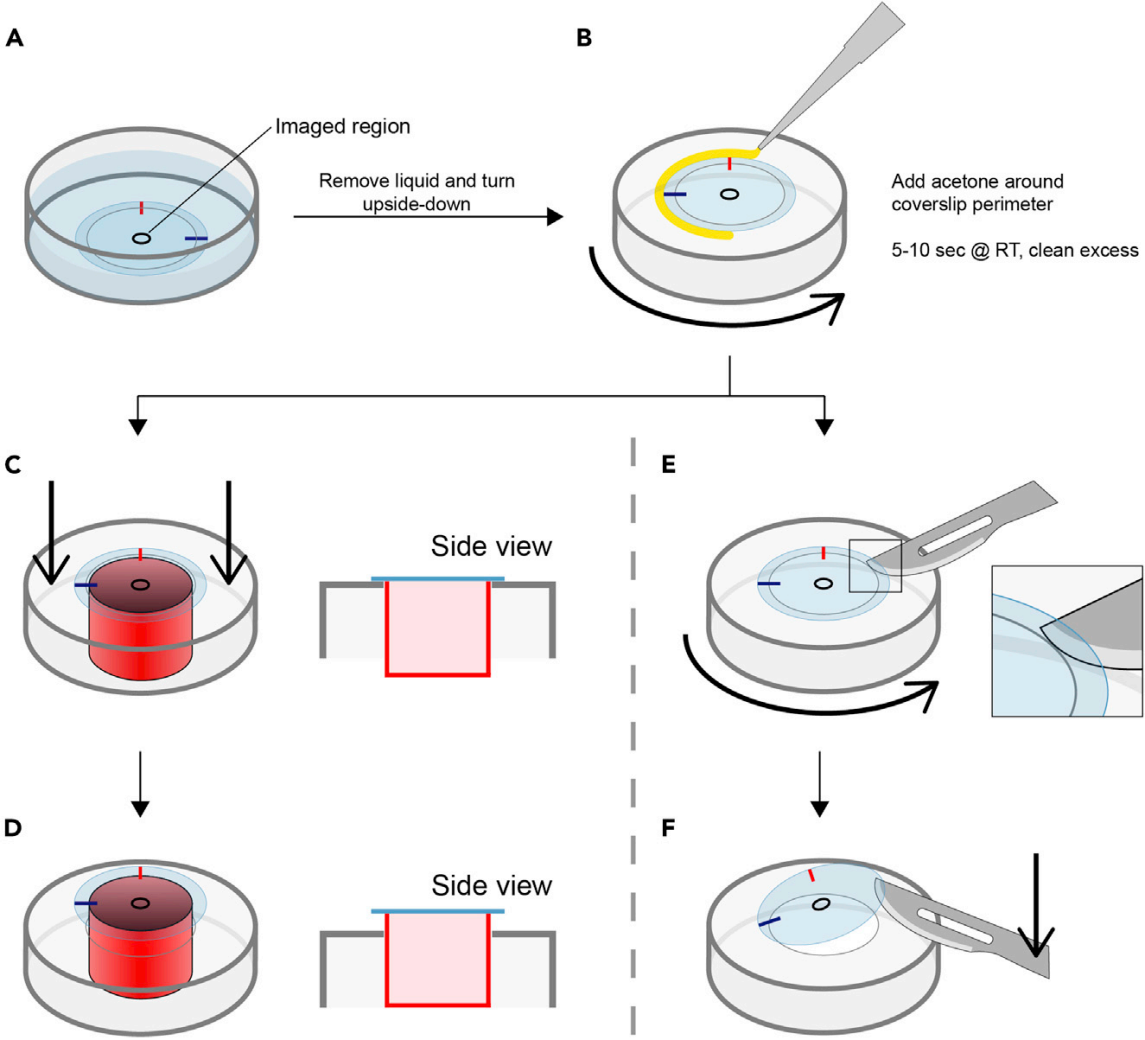
Coverslip extraction procedure (A) First, imaged region is marked on the bottom of the coverslip. (B–F) (B) Following buffer removal, the dish is turned upside-down, and acetone is added around the perimeter of the coverslip. Once the adhesive has softened, coverslip is extracted by uniformly applying pressure from the sample-side on the coverslip with a hollow cylinder (C and D) or by using a scalpel (E and F).
21. Now that the adhesive is soft, apply pressure homogeneously along the coverslip perimeter from the inside (sample side). This can be done by pushing the dish against a hollow cylinder with a slightly smaller diameter than the inset (Figures 3C and 3D).

***Note:*** This coverslip detaching technique involves touching the periphery of the sample surface, and must be carried out with extreme care.

20. Remove most of the liquid from the dish, turn it upside-down and add some drops of acetone around the perimeter of the coverslip. Incubate for 5–10 s (Figures 3A and 3B), then and clean any remaining acetone.

**Alternative:** If a hollow cylinder of the appropriate size is not available, or the nature of the sample is incompatible with the aforementioned technique, the coverslip can also be detached with the help of a scalpel. For this, Introduce a scalpel in between the coverslip and the plastic and gently rotate the dish to liberate the coverslip (Figures 3E and 3F). This technique, however, is prone to coverslip cracking and must be carried out with extreme care.

22. Once the coverslip is detached, place it (sample facing up) quickly on a 6-well plate with phosphate buffer and remove any remaining adhesive if needed.

### Dehydration and drying

**Timing: 10 h**

***Note:*** This protocol has been optimized for the acquisition of high resolution SEM images, which requires high vacuum conditions. Therefore, the samples must be dehydrated and dried before the session.

Sample dehydration is carried out in order to substitute all the water in the sample by an organic solvent (ethanol), preparing it for subsequent drying steps (Katsen-Globa et al., 2016). Ethanol is substituted then by hexamethyldisilazane (HMDS), a low tension volatile non-polar solvent that mediates sample drying without requiring any special equipment.

**CRITICAL:** These steps must be carried out on a fume hood. HMDS is highly toxic and must be handled wearing the appropriate personal protective equipment.

23. Immerse the samples in increasing concentrations of ethanol starting with 10%, increasing with 10% increments up to 100%, incubating 3 min per solution.
24. After reaching 100% ethanol, incubate 3 min in a 1:1 mixture of ethanol and HMDS, followed by an additional 3 min incubation in pure HMDS.

**CRITICAL:** Increased incubation times with HMDS can cause severe cell shrinkage.

25. Remove all the liquid from the dish and air-dry on the fume hood overnight.

***Note:*** This protocol has been optimized for epithelial cells, non-adherent cell types might require additional steps, such as osmium post-fixation.

**Pause point:** Uncoated biological samples might re-hydrate or degrade after drying. We therefore recommend carrying out these steps within one week before SEM imaging session. In the meantime, samples must be stored in a dry environment to help preserving their integrity.

### SEM imaging

**Timing: 2–5 h**

This step includes a first stage where the candidate cell surface features imaged by confocal microscopy are located. This is followed by a second phase where small-FOV SEM images of those structures are acquired.

**Figure 4:**
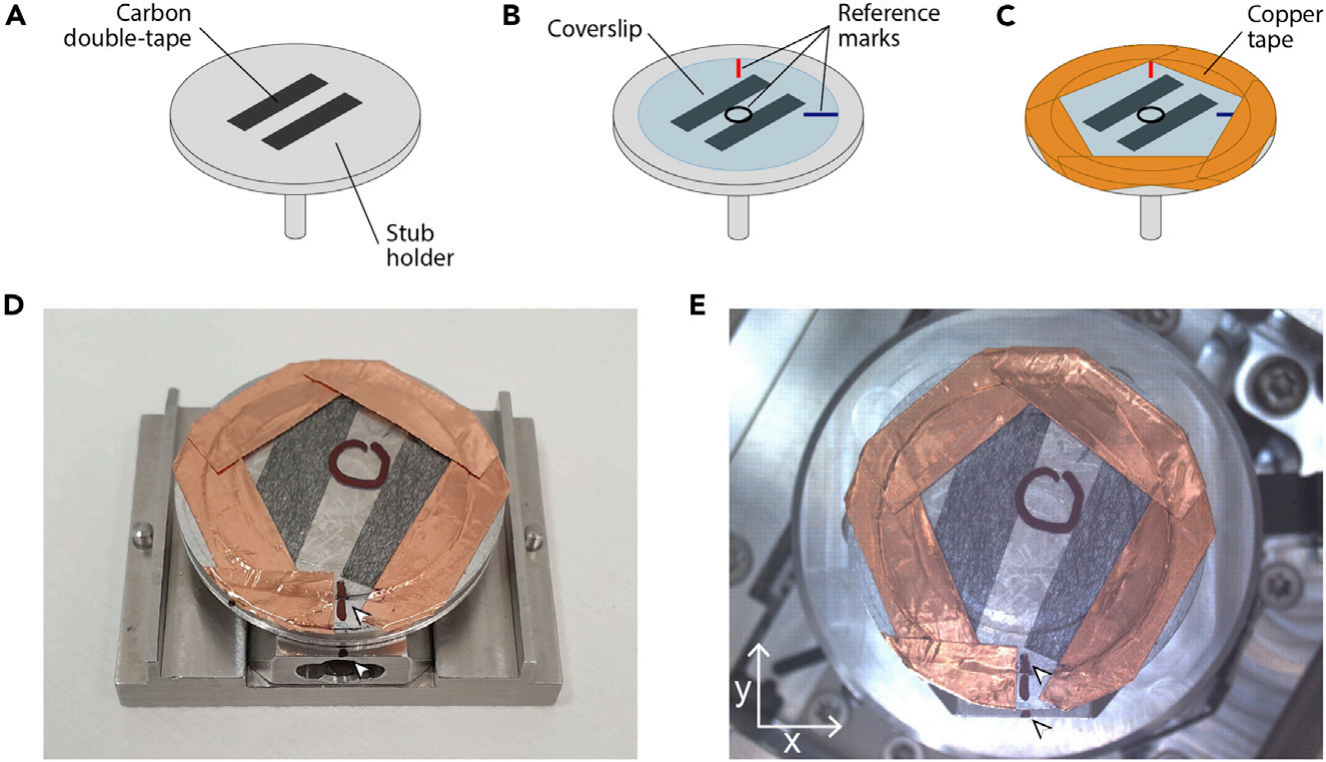
SEM sample mounting (A and B) Carbon double-tape is used to attach the coverslip to the stub holder. (C) Copper tape is used to provide some electric contact between the sample surface and the stub holder. (D) Sample is then placed on the holder, aligned to its axis using the reference marks (arrowheads) and introduced on the SEM chamber. (E) An in-chamber image can be used for navigation purposes, reference marks (arrowheads) can be used to align the sample with the microscope moving axis.
  b. After that, frame the edges of the glass substrate with metallic tape (copper, aluminum) (Figure 4C). In this way, the upper surface of the substrate is in electric contact with the SEM holder and this slightly helps to mitigate sample charging during imaging. Be careful not to cover or touch the area imaged by confocal microscopy.
  c. Place the stub on the SEM stage holder (Figure 4D) and introduce it on the SEM chamber. Orient the sample matching the microscope motion axis by using the reference marks.
  d. Keep track of the position of the imaged area (marked at the end “Confocal imaging” stage [step 17]) relative to an easy to identify part of the sample, such as the border.

***Note:*** Some SEM microscopes include optical navigation systems that allow to use an image of the mounted sample as a navigation reference (Figure 4E).

**Figure 5:**
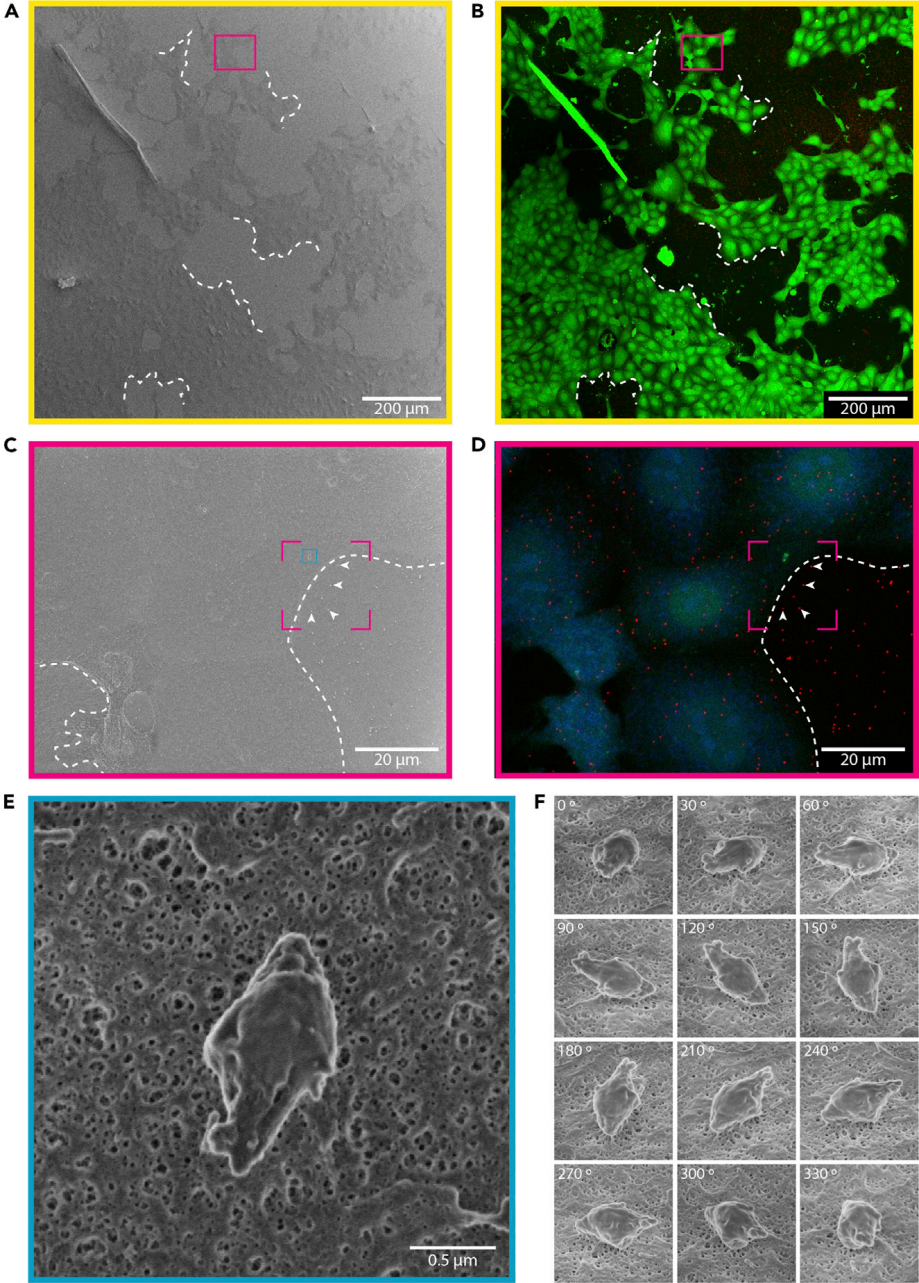
SEM large-, medium- and small-FOV images and initial alignment Large FOV SEM image (A) is compared to its confocal counterpart (contrast-enhanced) (B). The contours originated by the cell colonies (dotted lines in all panels) serve as navigation cues and allow to unequivocally find the same imaging area using different imaging techniques. Occasional imperfections in the sample, such as the dirt fiber shown in the top-left of A and B panels, can also facilitate orientation and alignment process. This allows the localization of the mid-FOV imaging areas (C and D) (magenta boxes on panels A and B) containing the structure of interest. The pattern formed by the Au NPs on the substrate (arrowheads on panels C and D) is used to localize the structures of interest on the SEM (blue box in C). Once the surface feature of interest has been localized on the mid-FOV SEM image, the small-FOV (E) and tilt images (F) can be acquired. Magenta dotted boxes in C and D correspond to the regions aligned in Figure 6 and represented after alignment on Figure 7.
  d. Compare both confocal and SEM large-FOV images and identify relevant structures such as the pattern formed by the cells, occasional defects, etc., that allow correlating them (Figures 5A and 5B). Correct the position and the orientation of the sample if needed. Typically, Au NPs are still not visible at this stage and therefore they cannot serve as a reference yet.
  e. Using the look-up map, start identifying the sections of interest in the sample. Acquire medium-FOV (100 μm wide, around 1,000×) images (Figure 5C). Dwell times in the range of 100–300 ns are appropriate here. The imaged area should be equivalent to the medium-FOV confocal microscopy images obtained in step 15 (Figure 5D). Areas of the substrate showing the Au NPs must remain visible. For the medium-FOV images the in-lens secondary electrons detector is preferable to the in-chamber one as it provides better resolution.
    ***Note:*** Depending on the size of the structures of interest, higher resolution images, in terms of pixels, might be needed at this point. As a reference, we employed 3072∗2048 pixels for the medium-FOV images, keeping the rest of the images at 1536∗1024 pixels. Image definition here must be high enough to allow the observation of the Au NPs and the identification of the surface features of interest by using the position of the Au NPs as a reference.
  f. By comparing confocal and SEM medium-FOV images, identify the regions where the structures of interest are located (Figures 5C and 5D). Then, the pattern formed by the Au NPs is used to univocally localize them.

26. Mount the sample on the SEM stub (large stub, diameter > 30 mm, see materials and equipment). Keep track of the substrate orientation and position of interest, as marked after confocal imaging:
  a. Use a thin conductive double sided tape (carbon) to attach the sample (cells on the upper side) to the aluminum stub (Figures 4A and 4B). Align the reference marks done on the sample during the last stage of “coating and addition of gold fiducial markers to the coverslip” section [step 6] with the stub in such a way that you can keep track of orientation.

27. Locate the surface features of interest identified by confocal microscopy:
  a. Navigate to the area imaged by confocal microscopy.
  b. Set the working conditions for the electron beam: desired working distance (we used 4 mm), very low voltage and low current to minimize charge effects. For the latter two parameters, we recommend 0.5–1 kV and 13–25 pA, respectively. Align the column.
    ***Note:*** when adjusting the working distance, consider that it must allow the acquisition of all the different FOV sizes required.
  c. Acquire a large-FOV (2 mm wide, around 50×) image in order to compare with the look-up map (Figure 5A). Either the in-chamber or the in-lens SE detectors can be employed here. Beam dwell times around 1–3 μs are usually adequate, as charge is not a big problem at this low magnification.

28. Small-FOV SEM image acquisition of the surface features of interest:
  a. Take small-FOV images (in the range of 10,000×−100,000×) with the in-lens SE detector. For imaging of midbody remnants, the range 25,000×−60,000× with FOV width of around 2–5 μm was employed (Figure 5E). At these high magnifications, low-dwell times (50–100 ns) will be mandatory to reduce charge effects. Frame integration (around 100–200 frames) with software drift correction activated will allow to increase the image signal-to-noise ratio.
    ***Note:*** Charge accumulation might be an issue at this magnification level. For persistent charge effect issues, see troubleshooting 4: Charge during SEM **imaging**.
  b. If views of the structures from different orientations are needed, tilt and rotate the sample stage (depending on the options available in your SEM equipment) and acquire images in the same conditions (Figure 5F).

**CRITICAL:** Intensive focusing on the structures can produce damage by the electron beam or induce the deposition of carbon coating. Therefore, careful alignment of the beam in a region close to the structure of interest is recommended before moving to the intended section. This way, the image is acquired without any previous exposition of the area of interest to the beam.

### Image alignment

**Timing: 1–2 h**

If the goal of the correlative approach is to localize a structure that stands out against the cell surface, the analysis of the overall pattern of the cells and the Au NPs (as exposed in the previous steps) is usually enough. If a more precise localization or correlation is required, confocal and SEM images must be aligned.

29. Open the medium-FOV confocal and SEM images on ImageJ, perform brightness and contrast adjustments if needed. Find the structure of interest and crop a region around it, including the closest Au NPs, in both images (magenta boxes in Figures 5C and 5D).

***Note:*** The closer the structure of interest is to the Au NPs, the better the alignment will be (see “troubleshooting 5: Image alignment” if this requirement is difficult to fulfill).

**Figure 6:**
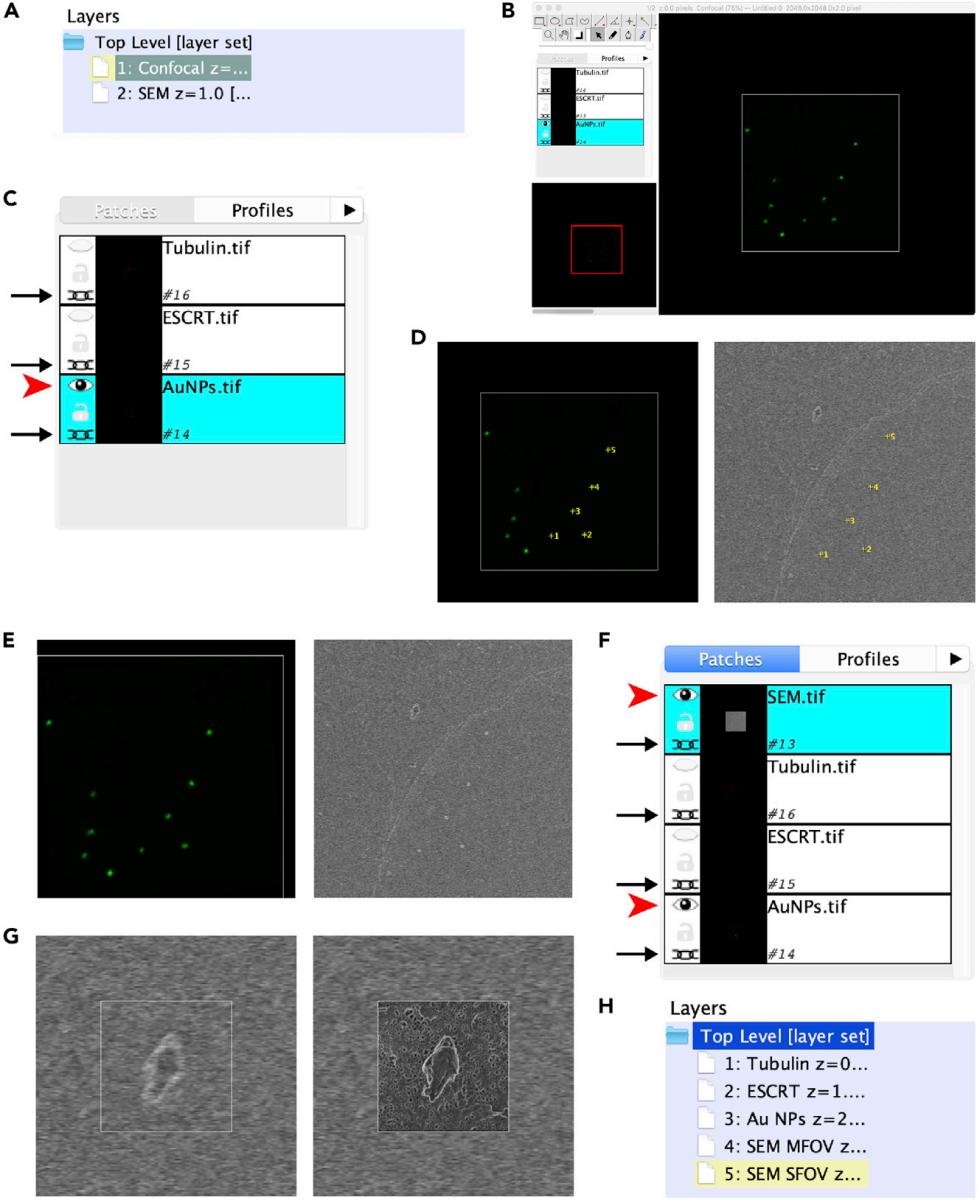
Alignment process with TrakEM2 plug-in (A) Detail of the “Project” window of TrackEM2, showing the “Layers” section including two layers for the confocal and SEM images respectively. (B) Overview of the “Navigation” window, including the “Patches” list in the left and an overview of the currently visible image (Au NPs in the example). (C) Magnification of the “Patches” list after confocal images have been imported, linked, and only the one containing Au NPs left visible. (D) Result of manual landmark registering in both confocal (left) and SEM images (right), note that the number assigned to each fiducial marker is the same in both images, and that their size is not the same due to different sampling. (E) Same images after alignment, note that the confocal image has been scaled and translated to match its SEM counterpart, which has remained unchanged. (F) Magnification of the “Patched” list after the inclusion of the SEM image in the same layer as the confocal counterparts, note that all the images are linked together to preserve the alignment. (G) Small-FOV SEM image is placed on top of the mid-FOV one and manually aligned. (H) Final state of the “Layers” section prior to stack exportation to ImageJ, note there is one layer per image in the dataset. Red arrowheads and arrows in C and F highlight the visible images and the linked ones, respectively.
  b. The “Navigation” window, consisting of a black canvas and surrounding menus (Figure 6B).

***Note:*** For further details on how to use TrakEM2 plugin, please refer to the manual available at www.ini.uzh.ch/∼acardona/trakem2.

30. Generate the final confocal image by selecting the desired planes, making projections, etc. Then, split the channels and save each one of them as a different TIFF file.
31. Start a new TrakEM2 project (File – New – TrakEM2 (blank)), two windows will appear:
  a. The “Project” window, formed by three columns (Figure 6A).

32. Create a new layer (on the “Project” window, layers column: select “Top Level [layer set]” - right click - new layer – z coordinate:1 – OK). Rename the layers as “Confocal” and “SEM” respectively (right click on each layer – rename) (Figure 6A).

**CRITICAL:** When creating a new layer on TrakEM2, a “z coordinate” value different than 0 must be chosen, otherwise the alignment process will fail.

33. Import all the confocal images to the “Confocal” layer (drag-and-drop the files from a folder to the canvas). The three images will be placed on top of each other, and will be listed on the “Patches” list in the left side of the window (Figure 6C). Move to the “SEM” layer (use “<” and “>” keys to navigate between layers) and add the SEM image to it following the same procedure.
34. Next, link all the images in the “Confocal” layer (so the different channels remain aligned) and make visible only the channel containing the Au NPs. This is achieved by using the “link” (chain) and “visibility” (eye) icons (Figure 6C).
35. Register both images using the Au NPs as manual landmarks (right click on the canvas – align – align layers manually with landmarks). Then, select the Au NPs near the feature of interest one by one in the same order (Figure 6D).
36. Align both images by “similarity” (right click on the canvas – apply transform – model: similarity – Propagate to first layer – OK), the layer containing the confocal image will be translated, rotated and scaled according to the Au NPs position (Figure 6E).

**CRITICAL:** The selected layer during the alignment process will be used as a reference, and will not be modified. In order to keep the orientation stable along the SEM dataset, the mid-magnification SEM image must be the one used as a reference.

37. Once both medium-FOV images are aligned (see limitations section), put them all in the same layer and link them (right-click on the SEM image in the “Patches” list – “Send to previous layer”) (Figure 6F).
38. Scale the images to match pixel size with the high magnification SEM image:
  a. Select all images from “Patches” list
  b. Right-click on canvas – transform – transform (affine)
  c. Right-click on canvas – specify transform. Introduce the same scaling factor in X and Y.
  d. Right-click on calvas – apply transform.

***Note:*** Pixel size can be found on the image metadata, or calculated considering the field of view and the resolution of the images. If necessary, re-scale the canvas to fit the entire image (Right-click – Display – Autoresize canvas/LayerSet).

39. Import the small-FOV SEM image to the same layer, and manually align it to the mid-FOV SEM image (Figure 6G). For this, image “visibility” icon (eye) can be used.
40. Create as many new layers as necessary, then move every image to a separate layer (select images on the “Patches” list – right-click – send to next layer) (Figure 6H).
41. Make sure all the images are visible and export the final stack to ImageJ (right-click on canvas – Export – Make flat image, set “Start” and “End” fields to span all the layers).

## Expected outcomes

**Figure 7:**
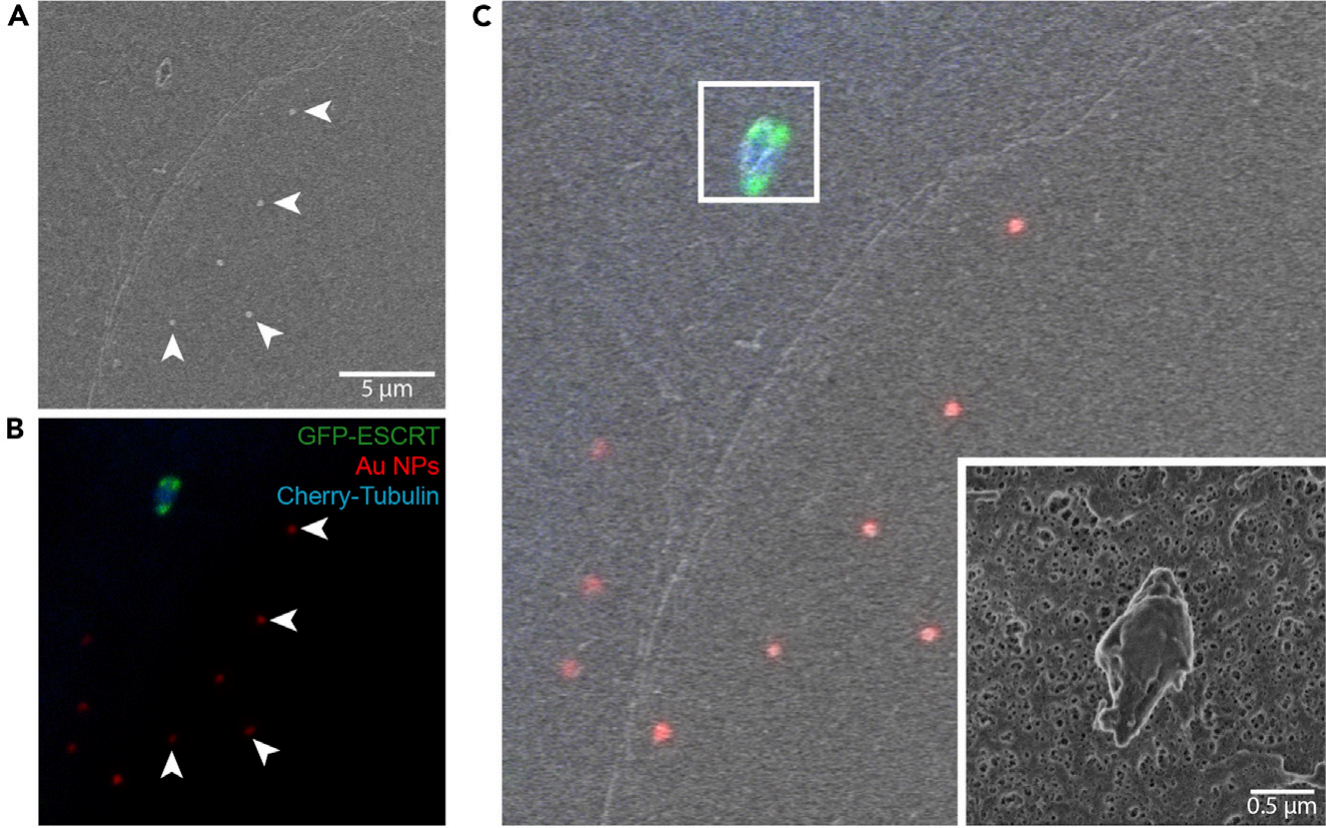
Expected outcome: correlated light and scanning electron microscopy images (A and B) The pattern formed by the Au NPs on the substrate (arrowheads) has been used to align both images, allowing their superposition (**C**). The position of the small-FOV image (C, inset) can be traced back to the original confocal dataset, this allows to combine the nanometric-range topographical SEM measurements with the multi-labeling protein localization data acquired through confocal microscopy.

After image acquisition, correlation, and alignment, the final result is an ImageJ hyperstack containing the medium-FOV confocal image aligned to the medium and small-FOV SEM images (Figures 7A–7C). This provides a topographical image of the cell membrane structure of interest with nanometric-range resolution, together with the multi-labeling protein localization data acquired through confocal microscopy. Additionally, and if tilt images were generated during SEM imaging, the feature of interest can also be observed from multiple angles (Figure 5F).

***Note:*** All the images included to illustrate this protocol (Figures 2, 5, 6, and 7) belong to the same dataset. The different FOV can be traced from one figure to another by using the following color codes: yellow for large-FOV, magenta for medium-FOV and blue for SEM small-FOV. A continuous box represents the entire FOV, whereas a dotted one corresponds to a cropped region.

## Limitations

Due to the different environmental conditions required for confocal and scanning electron microscopy imaging, dehydration and drying must be carried out in between both imaging methods (steps 23–25). It has been previously reported that drying of biological samples, regardless of the methodology used, decreases cell volume (Katsen-Globa et al., 2016). On a CLEM workflow, this results in a slight misalignment of the resulting images. Though fiducial markers over the substrate remain immotile, making alignment possible, the small movements of the cell surface due to shrinking could modify to some extent the localization of the structures of interest (see troubleshooting 5: Image alignment).

## Troubleshooting

### Problem 1

Using Alternative SEM without VLV capabilities

In this protocol, a scanning electron microscope capable of achieving subnanometric resolution under very low voltage (VLV) conditions has been used. This allows the analysis of uncoated biological samples on glass substrates, reducing sample manipulation and delivering the highest possible resolution. If a conventional SEM microscope without VLV capabilities is used, charging effects will appear during imaging (steps 27 and 28).

### Potential solution

Coating of the sample with a conductive material (such as gold, platinum or carbon) would minimize charging effects and allow the usage of higher beam voltages. However, this would be achieved at the expense of the level of detail in the final image, since the conductive layer will even out the finest structural details of the sample, potentially hiding relevant features. The coating should be done after sample dehydration and drying (subsequent to step 25). Deposition of a thin conductive coating, between 2 and 15 nm, by means of a physical vapor deposition technique should be enough. The thickness of the coating needs to be adjusted to allow the visualization of the Au nanoparticles.

### Problem 2

Use of confluent cultures

The proposed methodology for CLEM imaging strongly relies on the existence of large-scale patterns on the sample, such as the overall shape of the cell colonies, to localize the imaging area and perform an initial rough alignment. A confluent cell monolayer after the cell culture phase (step 9) looks homogeneous under low-magnification conditions, preventing the direct application of this protocol.

### Potential solution

In order to introduce a higher degree of physical confinement for the cells, while maintaining orientation cues inside the sample, micro-patterned substrates could be used.

Another possibility is the creation of a large-scale pattern on the sample after fixation by scratching out portions of the cell monolayer. This process, however, damages the cells right at the border of the pattern, which is the area where the alignment strategy works best.

### Problem 3

Confocal dataset tracing

Once the confocal large-FOV image has been acquired and the candidate features identified, the proposed methodology relies on the use of a motorized sample stage for repositioning during medium-FOV images acquisition (steps 15 and 16). In the absence of a motorized sample stage, or if the navigation cues present in the sample are too complex, this process might be challenging.

### Potential solution

In this case, the use of gridded coverslips (Mattek, Cat#P35G-1.5-14-CGRD) may be of help. The squared pattern embedded on the substrate, together with the alphanumeric code that identifies each square, allows to localize each medium-FOV image for look-up map elaboration. For this, an additional transmitted-light track has to be acquired during confocal imaging, as to make the pattern visible in the final images.

### Problem 4

Charging during SEM imaging

During SEM imaging (steps 27 and 28) of non-conductive samples, the accumulation of negative charges on the specimen surface causes strong contrast variations in the image or drift, precluding the obtention of good quality images.

### Potential solution

We recommend adapting the SEM imaging conditions as follows, with each step providing a higher level of complexity.

1. Keep the voltage at 1 kV but reduce the beam current.

2. Keep beam voltage at 1 kV and beam current at 13 pA (or lower beam current if desired), and apply beam deceleration (see materials and equipment section). A typical value for the sample bias to employ is 1000 V.

***Note:*** Beam deceleration strategies should be applied too if the image quality at 1 kV is too low.

**CRITICAL:** The use of beam deceleration strategies usually prevents the option of tilt imaging.

3. Reduce beam voltage at 500 V and keep beam current at 13 pA (or lower beam current if desired). Beam deceleration is usually mandatory in this case to obtain high resolution images at this very low voltage. A typical value for the sample bias voltage to be applied is 1500 V.

***Note:*** These softer conditions (options 1, 2 and 3) can also be useful for preventing damage of the structures.

### Problem 5

Image alignment

Dehydration and drying procedures carried out between confocal and SEM imaging (steps 23–25) may cause subtle deformations of the cell surface due to shrinking. Since the Au NPs are directly attached to the substrate, and thus unaffected by these deformations, there might be a slight shift in the position of the features of interest when confocal and SEM images are compared after alignment (step 41).

Additionally, the proposed methodology is optimized for the analysis of features that are relatively close to areas with exposed substrate, since alignment accuracy decreases with distance.

### Potential solution

Image alignment precision can be improved, and its application range extended by applying the Au NPs directly on top of the cell surface prior to fixation (step 10). This way, the Au NPs would be affected by the deformation of the cell surface at the same extent as the features of interest, allowing its compensation. This alternative approach would also allow the correlative analysis of features that are far from exposed substrate areas.

Though the vast majority of the protocol would be unaffected by this modification, the final image alignment process should be modified to include the movement of the Au NPs between imaging methods. While the proposed methodology uses only translation, rotation and scaling to achieve alignment (step 36), elastic deformation (“Affine” on TrakEM2) of one of the datasets might be needed to compensate the movement of Au NPs placed on top of the cells.

## Resource availability

### Lead contact

Further information and requests for resources and reagents should be directed to and will be fulfilled by the lead contact, Javier Casares Arias (javier.casares@bsse.ethz.ch).

### Materials availability

This study did not generate new unique reagents.

### Data and code availability

The published article includes all datasets generated or analyzed during this study.
